# Editorial: *In vitro* toxicogenomics (TGx) in hazard and risk assessment

**DOI:** 10.3389/ftox.2023.1284932

**Published:** 2023-09-05

**Authors:** Marc A. Beal, Logan J. Everett

**Affiliations:** ^1^ Bureau of Chemical Safety, Health Products and Food Branch, Health Canada, Ottawa, ON, Canada; ^2^ Biomolecular and Computational Toxicology Division, Center for Computational Toxicology and Exposure, Office of Research and Development, US Environmental Protection Agency, Durham, NC, United States

**Keywords:** TGx, toxicogenomics, *in vitro*, hazard risk assessment, animal toxicology

Toxicogenomics (TGx) involves the application of transcriptomics to study how cells and organisms respond to environmental and chemical exposures by measuring changes in gene expression. TGx technologies have proven to be valuable tools in human health and environmental risk assessment. Specifically, TGx data provides a wealth of mechanistic insight to support a weight-of-evidence approach when establishing links between exposure and adverse outcomes. Furthermore, short-term *in vivo* studies using animals as models have supported the derivation of transcriptomic points-of-departure (PODs), i.e., the dose levels expected to lead to chronic adverse outcomes, that serve as surrogates of PODs measured using traditional apical toxicological endpoints in longer-term studies (Thomas et al., 2013; Zhou et al., 2017; Gwinn et al., 2020; Johnson et al., 2020). However, given the concerted international efforts to reduce the use of animals in toxicity testing, it is anticipated that animal studies will become less frequent over time (Kavlock et al., 2018). Thus, there is a pressing need to build confidence in non-animal TGx approaches and promote alternative testing strategies that take advantage of higher throughput *in vitro* systems. This shift towards New Approach Methodologies (NAMs), including novel *in vitro* TGx methods, presents an opportunity to accelerate the pace of chemical risk assessment and establish a next-generation risk assessment strategy to protect humans and the environment from emerging chemicals of concern. This Research Topic presents a collection of articles highlighting recent *in vitro* TGx advancements to establish the utility of both TGx methods and data for hazard and risk assessment.

High-throughput transcriptomic biomarkers can predict specific adverse outcomes and establish PODs at which key molecular events occur following exposures. In the studies by Buick et al. and Fortin et al., a previously developed transcriptomic biomarker referred to as TGx-DDI (Li et al., 2015; 2017) was used to classify chemicals tested in human lymphoblastoid TK6 cells as either DNA damage-inducing (DDI) or non-DDI. Buick et al. investigated three anti-infective agents that had conflicting results in previous reports: nitrofurantoin (NIT), metronidazole (MTZ), and novobiocin (NOV). The TGx-DDI biomarker suggested that NIT and MTZ are unlikely to be DDI in human cells, while NOV was classified as DDI consistent with its ability to inhibit DNA topoisomerase II. Similarly, Fortin et al. used TGx-DDI alongside other genotoxicity NAMs in an *in vitro* only genotoxicity assessment approach for 10 data-poor chemicals. The incorporation of the TGx-DDI revealed detailed mechanistic insights that added to the weight-of-evidence to distinguish between chemicals that were likely irrelevant positives in the *in vitro* micronucleus assay, and chemicals that are weakly DDI. Combined, these studies demonstrate the utility of incorporating TGx biomarkers into *in vitro* genotoxicity assessments, particularly when conflicting results from other assays exist.

Read-across is a powerful and commonly used approach for chemical risk assessment to predict toxicity and mechanisms of action for target chemicals that lack experimental data, based on hazard data from structurally similar analogs. While guidance exists for read-across assessments based on traditional animal studies [e.g., (ECHA, 2017)], guidance for the integration of *in vitro* TGx data is lacking. The studies by Naciff et al. and Drake et al. demonstrate how TGx data can establish the suitability of structural analogs within chemical groupings and compare the bioactivity observed across chemicals to support read-across. Naciff et al. presented two case studies representing hypothetical read-across scenarios and demonstrated how an *in vitro* TGx read-across strategy could be used for data gap filling. One case study focused on four linear chain n-alkyl parabens, and the other focused on caffeine and its main metabolites. Four *in vitro* cell-based models (MCF7, HepG2, A549, and iCell Cardiomyocyte cells) were tested at varying concentrations for each chemical. Bioactivity results based on gene expression changes and pathway enrichment supported the use of the biological analogs for the read-across of target chemicals to support data gap filling. Similarly, Drake et al. presented a case study to support the use of *in vitro* TGx data in evaluating the mechanisms of action across grouped compounds. They examined a group of four α-diketones, three of which are suspected to induce pulmonary fibrosis in rodents, and tested the chemicals in primary human epithelial cells under air-liquid interface conditions. This case study also examined a structurally similar β-diketone and one γ-diketone to identify potential (dis)similarities. The analysis of gene expression data revealed that the four α-diketones displayed similar bioactivity levels, as demonstrated by the number of activated and shared pathways, and that the number of active signaling pathways decreased from α- to β- to γ-diketones. Together these studies demonstrate how *in vitro* TGx data can strengthen the plausibility of using analogs in the read-across of data poor target chemicals. As the risk assessment paradigm transitions to increasing use of NAM data, the bioactivity information provided by *in vitro* TGx data will be critical to the selection of analog chemicals to support the toxicity assessment of data poor target chemicals.

A major advantage of *in vitro* TGx data is that it provides a wealth of sharable information that can be re-analyzed to test new hypotheses. Tribondeau et al. leveraged previously published TGx data from mouse embryonic stem cells exposed to the flame retardant, tetrabromobisphenol A (TBBPA), to understand the molecular phenotype of TBBPA and discover unexpected effects on neuronal differentiation and on the immune system. Through their analysis, the authors also identified a set of candidate biomarkers to support neuronal and immunological toxicity assessment. Reardon et al. applied a uniform workflow across six TGx datasets, including concentration-response studies for 117 chemicals, three cell-based models, and diverse exposure durations to assess the strengths and weaknesses of current experimental designs. Furthermore, they derived transcriptomic PODs through benchmark concentration modeling and used *in vitro* to *in vivo* extrapolation to derive human relevant endpoints that are more practical for chemical risk assessment. Reardon et al. provided evidence that transcriptomic PODs are most often equal to or more protective than PODs derived from animal toxicity studies, and the different experimental approaches display a high degree of concordance. Together these studies demonstrate the robustness of *in vitro* TGx data and highlight the opportunities to use existing TGx data to explore new questions, leverage different analytical methods, and combine available datasets for meta-analyses.

In summary, this edition achieved its goal of presenting novel studies building confidence in the use of *in vitro* TGx methods and data for both hazard and risk assessment. The outcomes of the six studies included in the Research Topic serve as useful illustrations of how *in vitro* TGx data can be integrated into regulatory decision-making. The editors are grateful to all the authors and would like to thank them for their valuable contributions to both this Research Topic and the TGx field.
